# kmer-SVM: a web server for identifying predictive regulatory sequence features in genomic data sets

**DOI:** 10.1093/nar/gkt519

**Published:** 2013-06-14

**Authors:** Christopher Fletez-Brant, Dongwon Lee, Andrew S. McCallion, Michael A. Beer

**Affiliations:** ^1^McKusick-Nathans Institute of Genetic Medicine, Johns Hopkins University School of Medicine, Baltimore, MD 21205, USA, ^2^Department of Biomedical Engineering, Johns Hopkins University School of Medicine, Baltimore, MD 21205, USA and ^3^Department of Molecular and Comparative Pathobiology, Johns Hopkins University School of Medicine, Baltimore, MD 21205, USA

## Abstract

Massively parallel sequencing technologies have made the generation of genomic data sets a routine component of many biological investigations. For example, Chromatin immunoprecipitation followed by sequence assays detect genomic regions bound (directly or indirectly) by specific factors, and DNase-seq identifies regions of open chromatin. A major bottleneck in the interpretation of these data is the identification of the underlying DNA sequence code that defines, and ultimately facilitates prediction of, these transcription factor (TF) bound or open chromatin regions. We have recently developed a novel computational methodology, which uses a support vector machine (SVM) with kmer sequence features (kmer-SVM) to identify predictive combinations of short transcription factor-binding sites, which determine the tissue specificity of these genomic assays (Lee, Karchin and Beer, Discriminative prediction of mammalian enhancers from DNA sequence. *Genome Res.* 2011; 21:2167–80). This regulatory information can (i) give confidence in genomic experiments by recovering previously known binding sites, and (ii) reveal novel sequence features for subsequent experimental testing of cooperative mechanisms. Here, we describe the development and implementation of a web server to allow the broader research community to independently apply our kmer-SVM to analyze and interpret their genomic datasets. We analyze five recently published data sets and demonstrate how this tool identifies accessory factors and repressive sequence elements. kmer-SVM is available at http://kmersvm.beerlab.org.

## INTRODUCTION

Understanding the function of DNA regulatory elements in the human genome remains a significant challenge. These elements include enhancers, repressors and insulators, which regulate the expression of their associated genes, and are widely believed to play a significant role in human development, physiological homeostasis and disease. Recent genome-wide association studies have found that 80% of common human variants significantly associated with a phenotypic trait (*P* < 10^−^^8^) lie in intergenic or intronic regions (1), and a significant fraction is thus suspected to affect these regulatory processes. As a result, a major component of current research development is focused on developing a more complete understanding of the regulatory biology of genomes. Genome-wide assays of expression and transcription factor (TF)-binding are essential tools in these studies and have been greatly enabled by the development of massively parallel sequencing. Chromatin immunoprecipitation followed by sequencing (ChIP-seq) is now routinely used to identify genomic regions bound by a TF or co-activator in a specific cell-type or condition of medical interest. However, although these experiments generate large and reproducible data sets, determining the underlying molecular mechanisms, which specify the biological function of these regions, remains extremely difficult. For example, enhancer activity is modulated by the cooperative binding of clusters of TFs, which stabilize a complex of co-activator proteins that then modulate the activity of RNA Polymerase II at the gene promoter through direct contacts mediated by DNA looping. Chromatin accessibility contributes to the stability of this complex through histone-modifying activities recruited by the complex or established by prior events. Because of its central role in this regulatory process, many bioinformatic methods have been used to identify single TF-binding sites overrepresented in a set of genomic regions. *De novo* identification of putative position weight matrices (PWMs) from expression or binding data has met with some success, particularly in yeast (2), and this conventional motif finding can be successful when the set of genomic regions is small (in terms of total bp) or if the specificity of the TF is strong (e.g. high information content binding sites). Usually however, when conventional motif-finding approaches are applied to the high confidence set of genomic regions identified in a vertebrate ChIP-seq experiment, these approaches generate a large set of putative binding sites each with relatively weak predictive power, as will be discussed in greater detail later.

To bridge this gap, we have recently developed an alternative approach to predict enhancers using a complete set of oligomers or ‘kmers’ as features in a support vector machine (SVM) (3). This method, which we refer to as ‘kmer-SVM’, can accurately predict regulatory sequences without any prior knowledge about TF-binding sites. After training on an experimentally determined set of regulatory regions, each kmer receives a ‘weight’, which represents its overall contribution to enhancer activity. Our approach is significantly different from using a motif finder to generate a list of overrepresented motifs: our SVM finds the set of kmers, which in combination most precisely specifies the full set of bound genomic regions. Thus, the high weight kmers span the sequence features (e.g. the set of binding sites for the TFs) needed to specify the regions’ activity in the tissue or condition assayed.

In addition to cross-validation (CV) and the human and mouse examples detailed in ‘Results’ section, the predictions of our kmer-SVM have been independently experimentally validated. In our previous study (3), we trained a kmer-SVM on EP300 bound enhancers in embryonic mouse forebrain (4) and verified that our method predicted independently obtained DNaseI hypersensitive regions (5) in similar embryonic mouse tissue with 56.3% precision, despite the reduced specificity of the DNaseI assay for enhancers (DNaseI also detects other open chromatin regions). We have also recently successfully applied this method to identify and experimentally validate a predictive regulatory sequence vocabulary in melanocytes (6). Using a kmer-SVM trained on EP300 bound regions in mouse melanocytes, we identified 7361 additional putative enhancers. We subsequently analyzed 11 of these regions with luciferase expression assays and validated 73% of them as having significant *in vitro* enhancer activity in melanocytes, only slightly reduced from the validation rate on the enhancer training set. We further showed that at least two of three predicted enhancers assayed *in vivo* directed GFP expression in the melanocytes of mosaic transgenic zebrafish. Additional unpublished work has broadened these pilot validations both in enhancer prediction and by targeted mutagenesis of predictive kmer sequence features.

Here, we present a web server to allow users to independently perform this analysis on any set of DNA sequences identified in a genomic assay. We expect that this tool will aid experimenters in the analysis of their own genomic data sets in several ways. First, high kmer-SVM classification accuracy is an independent measure of the quality of the genomic data, and can be used, for example, to optimize normalization methods, thresholds and the treatment of biological replicates by systematically maximizing classification performance, as genomic data sets with reduced noise can be predicted with higher accuracy. Second, identifying recognizable TF-binding sites among the most significant positive kmer-SVM feature weights gives further confidence in the positive set of genomic regions. Third, the identification of unexpected TF-binding sites among the most predictive features frequently generates novel hypotheses for subsequent experiments. Fourth, the highest scoring kmers provide focused targets for mutations predicted to modulate enhancer activity in validation experiments. Finally, predictive kmer-SVM features can be used to prioritize targets among disease associated SNPs within larger haplotypes in linkage disequilibrium.

We have chosen to use the Galaxy platform (7,8) as a framework for our web server, but our tool can also be used as a standalone set of programs. Here, we describe its use and demonstrate how to use the kmer-SVM to extract useful information from data sets generated by high-throughput sequencing-based experiments.

## MATERIALS AND METHODS

### Overview of the kmer-SVM galaxy module

**Figure 1.**
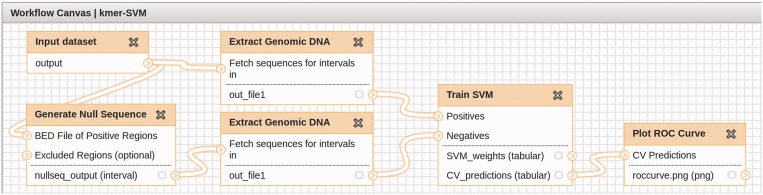
An example workflow for the kmer-SVM module. This workflow consists of three different components from the kmer-SVM module, ‘Generate Null Sequence’, ‘Train SVM’ and ‘Plot ROC Curve’ and one built-in Galaxy module, ‘Extract Genomic DNA’.

Our proposed analysis pipeline to identify regulatory DNA sequence features consists of three main components: (i) Generating the positive and negative sequence sets, (ii) training the SVM classifier and (iii) analyzing its performance and predictive sequence features. Although the positive training sequence set is provided by the experimenter in the form of a BED file of coordinates or sequence data in FASTA format, including genomic coordinates, the negative set is generated by our module ‘Generate Null Sequence’. SVM training is fairly transparent, takes the positive and negative sequence sets as input and produces a set of kmer weights and predicted class labels as output using CV. Finally, the performance of the SVM classifier is summarized by Receiver Operating Characteristic (ROC) and Precision-Recall (PR) curves, and features are ranked by their significance. Figure 1 shows the general workflow generated by Galaxy’s ‘Workflow Editing Tool’. This figure uses the actual Galaxy module names and data files, and this workflow can also be used as a template for a typical analysis pipeline.

### Details of core modules

#### Generation of sequence sets

Our kmer-SVM classifier takes as training data a FASTA file of positive sequences obtained through ChIP-seq, DNase-seq or another experimental assay, and a negative sequence set. To ensure that the SVM identifies sequence features specific to the positive regions, it is essential to match the GC content, length and repeat fraction when constructing the negative set, otherwise sequence features could be predictive simply by their enrichment or absence in the biased negative set (Supplementary Table S1). We refer to the set of the three distributions of GC, length and repeats in the positive set as its ‘sequence profile’ and the ‘Generate Null Sequence’ module matches this sequence profile for the negative set by using the following random sampling procedure. First, a positive sequence is randomly selected, and ‘Generate Null Sequence’ samples the same chromosome for a match in terms of length, GC content and repeat fraction, which does not overlap any positive sequence or existing negative sample by even one base pair. This random selection process is then repeated until the negative set has reached the requested size. This random selection process uses a precomputed table of genomic indices that are currently provided for the *Caenorhabditis elegans*, *Drosophila melanogaster*, mouse and human genome. The full negative sequence set then by construction closely approximates the sequence profile of the positive set. In special cases, users can exclude regions other than the input positive sequences from consideration for negative sequence generation through the ‘Excluded Regions’ option. We recommend using a negative set which is larger than the positive set, as doing so generally improves the statistical robustness of the classifier (Supplementary Table S2). We allow the user to specify the size of the negative set as an integral multiple of the number of positive sequences (say 10×) in the ‘# of Fold-Increase field’. As some positive sequences may not have exact matches in terms of GC content or repeat fraction, users can specify the percentage of GC content or repeat fractions by which a generated null sequence may differ from its corresponding positive sequence. This additional flexibility speeds the generation of the negative set and affects how precisely the negative set sequence profile matches the positive set sequence profile. Also, distinct realizations of null sequence sets may be generated by varying the ‘Random Number Seed’ parameter. The output of the Generate Null Sequence tool is a BED file describing the coordinates of the negative genomic intervals.

After the coordinates are specified, the actual sequences needed for SVM training are generated from the positive and negative BED file coordinates by the built-in Galaxy tool: ‘Fetch Sequences’, whose output is FASTA format DNA sequence files.

#### SVM training

An SVM (9,10) is a classifier, which attempts to find a hyper-plane boundary in feature space that separates elements of the positive and negative sequence sets. SVMs use techniques known as ‘kernels’ (9), which allows to define similarities between any two data points without explicit mapping of the data into a higher-dimensional feature vector space. A set of kernels called ‘string kernels’ have been developed for analyses of sequence data sets and have achieved great success in computational biology (11). ‘Train SVM’ uses one of these string kernels, specifically, the spectrum kernel (12). In our model (3), the features are the complete set of kmers, and their frequencies are calculated from the input FASTA files. The training module ‘Train SVM’ generates the normalized kmer count vector for each sequence and then finds the SVM internal parameters (support vectors) that most accurately distinguish the positive and negative sets. Currently, ‘Train SVM’ supports two kernels: the spectrum kernel (using a single length kmer) and the weighted spectrum kernel (using a user specified range of k’s, with equal weighting). In both cases, reverse complement kmers are treated as separate instances of the same feature. This module was implemented using the SVM Shogun toolbox (13).

‘Train SVM’ performs two tasks: it generates a set of ranked kmer-SVM weights, and it generates a set of class predictions using CV. A given kmer’s score can be thought of as a measure of the degree to which that kmer contributes to the discriminatory power of the classifier. The weights are output to the table labeled ‘Weights’.

#### CV

As is standard in machine learning, CV is used to assess classifier performance. The initial positive and negative sets are randomly partitioned into *n* distinct sets (for *n*-fold CV), and the ROC and PR performance of each test set is generated using a classifier trained on the other *n*-1 sets. The number of CV sets is a parameter, which can be specified by the user. This is repeated for all *n* partitions such that in the end each partition is used for both training and test-set scoring. The result of this process is the set of scores for test-set sequences in each round of CV, output in the table labeled ‘Predictions’.

Three parameters for SVM learning are adjustable (*k*, *C* and *E*). If the spectrum kernel is used, *k* specifies a single kmer length, whereas if the weighted spectrum kernel is used, minimum and maximum values for *k* must be set. Using a single *k* is somewhat easier to interpret in the beginning, as the vocabulary is simpler. Using a range of *k* values does have the advantage that similar kmers of slightly varying length and composition should all receive significant weights, increasing confidence in interpretation. Also using a range (e.g. 5–8) usually performs incrementally better than a single *k* in terms of overall classification accuracy.

The SVM maximizes the margin between the positive and negative sequences while simultaneously minimizing errors (sequences on the wrong side of the boundary). The relative importance of misclassification error is weighted by the regularization parameter, *C.* In practice, this affects over-fitting. A small *C* will result in less over-fitting of the SVM at the expense of slightly greater training classification error, whereas a large C will result in more over-fitting of the SVM (Supplementary Table S3). With unbalanced positive and negative set sizes, it is often recommended to use a separate regularization parameter for positive and negative sequences, reflecting the relative importance of errors. We specify this using an additional parameter *Positive_Set_Weight* or *PSW*. The regularization parameter for the positive set is *C* * *PSW*, whereas for the negative set, it is *C*. The default setting is *PSW* = 1 + log(*N*/*P*), which weighs positives more heavily when the negative set is large. The rationale behind this formula is our observation that optimal *PSW*s usually follow the logarithm of the ratio between positives and negatives (Supplementary Table S4). In practice, results are insensitive to *C* and *PSW* unless there is a significant imbalance between the positive and negative set sizes. Finally, the precision parameter *E* constrains the precision of the SVM classifier. Increasing *E* results in a reduced number of support vectors and can lead to a more robust classifier by reducing the requirements on the accuracy of the classifier on the training set (14). In practice, the results should be insensitive to the choice of *E*, and the default value is recommended (Supplementary Table S5).

Example runtimes are provided in Supplementary Table S6 for kmer-SVM training for several combinations of sizes for positive and negative data sets, from a positive data size of 1000 sequences and an equivalently large negative data set to a positive data set of 10 000 sequences and negative data set of 100 000 sequences. Briefly, using the ‘Select Random Lines’ tool default to Galaxy installation and setting a random seed of 1, either 1000 or 10 000 intervals were randomly selected from each sample’s BED file. Negative sequences were generated using kmer-SVM’s ‘Generate Null Sequence’ tool, again with random seed of 1. Runtime increases as a function of the total number of sequences in the positive and negative data sets, and ranges from under 1 to 40 min for the data sets listed.

#### Interpretation of kmer SVM weights

The output of SVM training is a list of kmer weights, and it is the weighted sum of normalized kmer counts in a sequence that determines the predicted class. In biological terms, the presence of kmers with large positive weights significantly increases a sequence’s likelihood of being positive (e.g. being an enhancer or being bound by a TF in a specific cell type). Large negative weights are equally informative, as their absence significantly increases the probability of being positive [e.g. a binding site for a transcriptional repressor (3)]. The weights file output by ‘Train SVM’ lists all kmers and their corresponding scores. The SVM weight is a continuous valued quantity, and large absolute value is a direct measure of significance. It is the scores with large absolute values that will be of particular value to the biologist. The TFs binding the highest and lowest scoring kmers, if previously studied, can be found using database matching programs such as TOMTOM (15), using the UniPROBE, TRANSFAC and JASPAR databases (16–18). Finding the best PWM match to a kmer does not necessarily imply that that factor binds the kmer in the given context because many TFs have overlapping specificities, and the PWM databases are far from complete. However, we have found that large positive scoring kmers are often recognizable as TF-binding sites known to be important in the cell type of interest, whereas large negative scoring kmers have identified an important role for repressors in previously unknown contexts (3).

### Classification performance analysis

The area under the ROC curve (AUROC) and the PR curve (AUPRC) are measures of the accuracy of the classifier. AUROC corresponds to the probability that a randomly selected positive sequence will score higher than a randomly selected negative sequence. For each possible SVM score threshold, we calculate the true positive rate [*TPR* = *TP*/(*TP* + *FN*), or sensitivity] and false positive rate [*FPR*=*FP*/(*FP*+*TN*), or 1-specificity] at this threshold, where TP is the number of true positives, TN is the number of true negatives, FP is the number of false positives and the FN is the number of false negatives. The ROC curve plots *TPR* versus *FPR*. The PR curve plots Precision versus Recall, where, *Precision = TP*/(*TP + FP*) and *Recall = TPR.*

The ROC and PR curves are slightly different measures of the classification performance of the trained SVM: the ROC emphasizes true and false positive rates, whereas the PR curve emphasizes true positive predictions. This difference results in the ROC possibly overestimating the accuracy of a classifier for data sets with large imbalances in the positive and negative class sizes, as is typical of genomic predictions with large negative sets. The PR curve is more appropriate in the case of large negative sets, yielding more accurate evaluations of classifier performance because it directly assesses the accuracy of positive predictions.

### Details of auxiliary modules

#### Score sequences of interest

Once the SVM is trained, in addition to classifying the CV test sets, it can be used to score any sequence of interest. An additional detail is that although the rank of the SVM scores is significant, the scale of the SVM scores is not. We therefore turn this SVM score into a probability that the element is positive, by reporting the posterior probability that each sequence is in the positive class, using the algorithm described in (19,20). ‘Score Sequences of Interest’ takes as input a set of sequences in FASTA format and outputs the SVM score and posterior probability. Parameters to produce this posterior probability are included in the weight table output by ‘Train SVM’. ‘Score Sequences of Interest’ can also be used to make genome-wide predictions using the module ‘Split Genome’, which splits a genome into chunks of a length *c* bp that overlap each other by *v* bp. The results of ‘Split Genome’ can then be used as input for ‘Score Sequences of Interest’.

#### Sequence profiles

As discussed earlier in the text, the sequence profiles, or distributions of length, GC content and repeat fraction content in the positive and negative sequences are matched by ‘Generate Null Sequence’. It may be useful to compare the sequence profiles of other sets of genomic intervals; therefore, we have provided an additional module to perform this analysis. For a given BED file, this module calculates and reports the sequence profile of the regions specified by these coordinates.

#### Kmer to MEME

This utility module takes the output file of weights created by training a kmer-SVM and generates PWMs for kmers with the largest and smallest (most positive and most negative) weights. The user specifies how many kmers to be returned, with a maximum of 50. The output of this program is a MEME-formatted (21) list of PWMs.

#### Tomtom

To enable users to visualize the kmers identified as predictive by kmer-SVM, we also have implemented a local instance of the Tomtom (15) program. Briefly, Tomtom searches databases of TF motifs for matches with input motifs by using column-wise similarity measures between PWMs. Users can create PWMs using our ‘Kmer To MEME’ tool and use this as input for Tomtom. For measures of similarity, we offer the Euclidean distance, which can be thought of as the length of the straight line between two PWMs, the Pearson correlation coefficient, which measures the similarity between two PWMs, and the Sandelin–Wasserman function (22), which sums the column-wise differences between PWMs. We also offer the choice of *E*-value or *q*-value (23) as scoring criteria. The *E*-value controls the expected number of false positives and can be any number, whereas the *q*-value controls the false discovery rate and is a number between 0 and 1. Most users are advised to run Tomtom in the default configuration of the Pearson correlation coefficient as distance metric and the *q*-value as criteria. At this time, we only offer text output from Tomtom.

### Tutorial

We offer a tutorial on kmer-SVM, and the Galaxy interface generally, to introduce users to our software. Our tutorial walks users through navigating the Galaxy interface and then shows the steps of a typical kmer-SVM workflow. Our tutorial is at http://kmersvm.beerlab.org/tutorial/ and can also be reached from the kmer-SVM homepage.

### Software availability

kmer-SVM is available to users through a variety of channels. Primarily and for greatest ease of use, we offer a web server, located at http://kmersvm.beerlab.org. Additionally, the kmser-SVM suite can be downloaded from the Galaxy Toolshed (http://toolshed.g2.bx.psu.edu/) by visiting the Toolshed and searching for ‘kmer-SVM’ and can be installed locally together with the Galaxy project framework. Supporting files for kmer-SVM suite, a list of dependencies required to run kmer-SVM suite, as well as an installation guide, can be found at http://kmersvm.beerlab.org/install/. Additionally, this information and further documentation is also provided as part of the kmer-SVM download (see README.txt).

## RESULTS

### Prediction of estrogen-related-receptor beta bound regions in mouse ES cells

**Figure 2.**
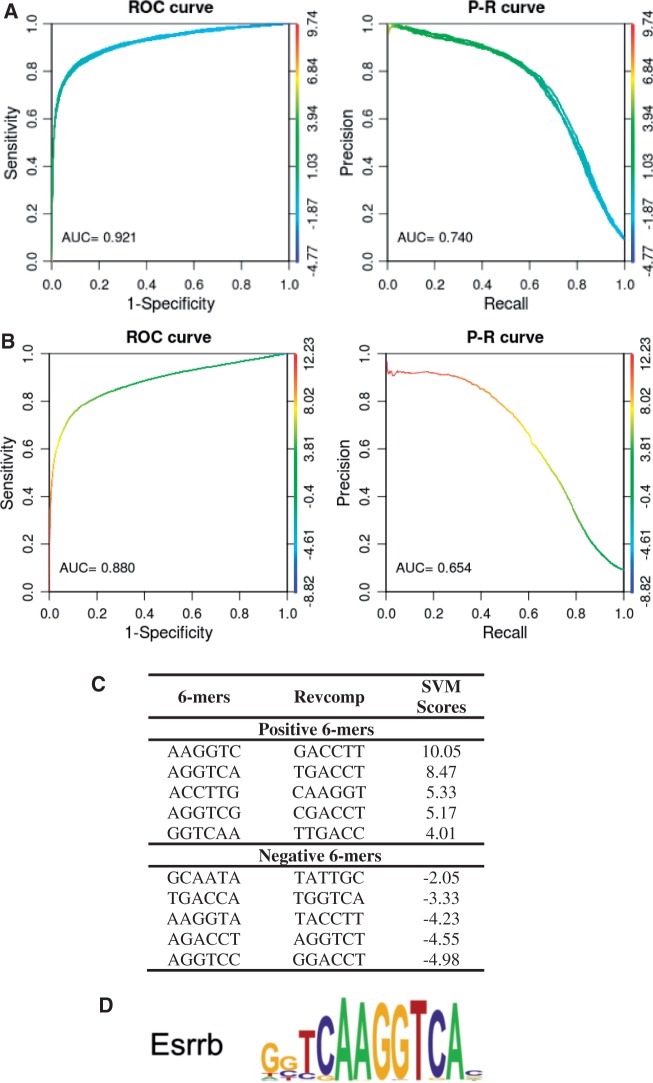
kmer-SVM analysis of ESRRB-binding sites. (**A**) ROC and PR curves for a kmer-SVM trained on ESRRB-bound genomic loci in ES cells versus 10-fold larger random genomic sequence. Default parameters were used for this analysis; Kernel type = Spectrum, *K* = 6, *C* = 1, *E* = 1e-5, *PSW* = auto. (**B**) ROC and PR curves for the ESRRB PWM scores. (**C**) The top five positive and negative 6 mers recover the ESRRB motif (**D**) reported in Chen *et al.* (24).

To take a specific example, we first consider the ChIP-seq data set of Chen *et al*. (24), who identified binding loci of TFs in mouse embryonic stem (ES) cells. As an example, we analyze their ChIP-seq data for estrogen-related-receptor beta (ESRRB) known to play a role in maintaining the pluripotency of ES cells (25). Because the ESRRB bound regions reported by Chen *et al*. (24) were short (10–30 bp), we extended from the midpoint of these regions and used 100 bp elements as the positive sequence set. Following the workflow in Figure 1, we then used ‘Generate Null Sequence’ to produce a 10× negative set, trained the SVM, then generated the ROC and PR curves for Chen’s ESRRB data set as shown in Figure 2a. These curves are typical of an accurate classifier, and we obtained summary statistics of AUROC = 0.921 and AUPRC = 0.74 for this data set. To directly compare the kmer-SVM prediction results with the PWM scores, we calculated the maximum log-odd score of the ESRRB PWM for each sequence and then plotted the ROC and PR curves as shown in Figure 2b. Although the ESRRB PWM is regarded as an easy motif, its classification performance (AUROC = 0.88 and AUPRC = 0.654) is significantly lower than kmer-SVM.

The top five positive and negative kmers reported by ‘Train SVM’ are shown in Figure 2b. Also in Figure 2c for comparison is the PWM for ESRRB found and reported in Chen *et al*. (24). As expected, the top kmers span the core motif of the ESRRB-binding site, but interestingly, several SVM-predicted kmers contribute to the specificity of the ESRRB. For example, AAGGTC (first), AGGTCA (second), CAAGGT (third), AGGTCG (forth) and so forth have large positive weights, but AGGTCC and AGGTCT have large negative weights, showing that A or G is allowed in the binding site at the 11th position of the PWM, but that C and T are not. This subtlety is not reflected in the PWM found by Weeder, the motif discovery algorithm used in Chen *et al*. (24).

### Prediction of distinct Glucocorticoid receptor bound regions in 3134 and AtT20 cells

We next show how our kmer-SVM can be applied to identify sequence features responsible for directing the binding of a single TF to different genomic locations in distinct tissues, developmental states or cell lines. As an example, John *et al*. (26) investigated the genomic binding of the Glucocorticoid Receptor (GR) TF in response to hormone stimulation in two divergent cell lines. Specifically, GR binding was profiled via ChIP-seq on a mouse mammary adenocarcinoma derived cell line (3134) and mouse pituitary (AtT20) cells. The binding of GR in these two cell lines were largely at non-overlapping genomic loci. John *et al*. (26) showed that the consensus GR-binding element (GRBE) was present in both 3134 and AtT20 bound regions, but that distinct sets of accessory sequence motifs were detected in the two cell lines, including binding sites for AP1, AML1, HNF3, TAL1 and NF1.

**Figure 3.**
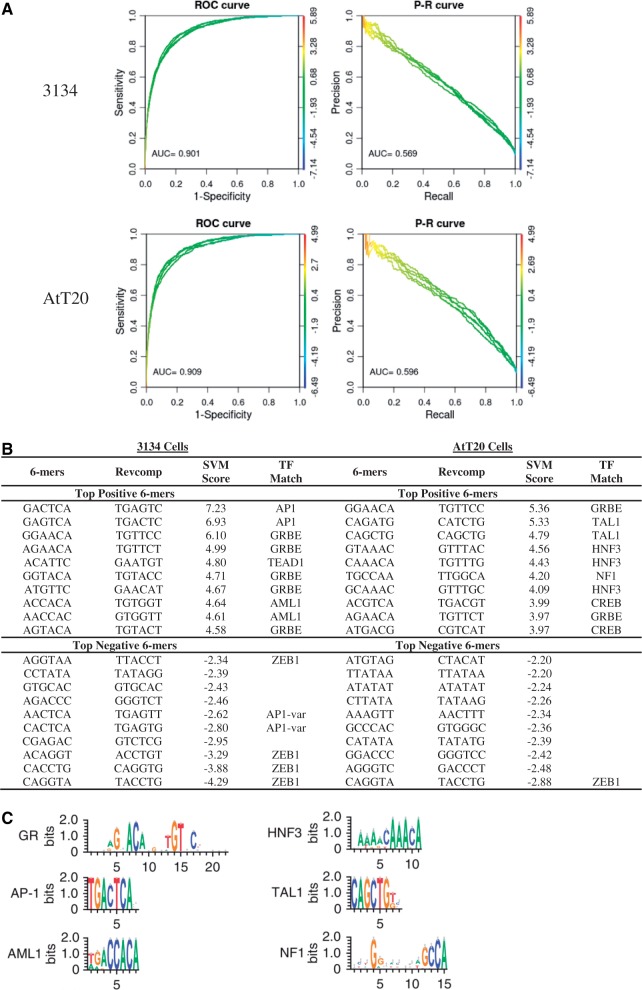
kmer-SVM analysis of GR-binding sites. (**A**) ROC and PR curves for a kmer-SVM trained on GR bound loci in 3134 cells and AtT20 cells versus 10-fold larger random sequence. Default parameters were used; Kernel type = Spectrum, *K* = 6, *C* = 1, *E* = 1e-5, *PSW* = auto. (**B**) The 10 most positive and negative 6 mers from 3134 cells and AtT20 cells recover the previously reported GRBE, AP1, AML1, HNF3, TAL1 and NF1 motifs (**C**) from John *et al.* (26), and additional novel accessory factors: CREB, TEAD1 and ZEB1.

We followed the Galaxy pipeline described earlier in the text to train a kmer-SVM on the ChIP-seq GR bound loci in 3134 cells versus 10× random genomic sequence and separately on GR bound loci in AtT20 cells versus 10× random genomic sequence, using the coordinates in John *et al*. (26) as positive set input. Our kmer-SVM classifier achieved an AUROC of 0.901 and AUPRC of 0.569 in 3134 cells, and AUROC of 0.909 and AUPRC of 0.596 in the AtT20 cell line (Figure 3a), indicating that GR binding in both cell lines is predictable based on sequence. The top 10 positive and negative weight kmers for each cell line are shown in Figure 3b, recovering kmers that span the GRBE and binding sites for accessory factors reported in John *et al*. (26). Although high scoring kmers matching the GRBE consensus were found in both cell lines, the accessory factors are specific to each cell line. In 3134 cells, the top two ranking kmers both match AP-1, and the eight and ninth highest kmers in 3134 cells matched AML1. Our kmer-SVM also identified TEAD1 as the fifth most important kmer (ACATTC), a binding site not found in John *et al*. (26). In addition, four of the most negative kmers match the binding site for ZEB1 or Snail, a common negative sequence feature in our analysis (3), indicating that the absence of ACCT or AGGT is predictive for GR bound regions. Thus, we hypothesize that either the presence of a ZEB1-binding site would directly inhibit the binding of GR, presumably through the binding of ZEB1 or another factor that binds specifically to this site. In other cases, this binding site could otherwise disrupt the normal function of the enhancer elements and is thus required to be absent (3).

In the AtT20 cells, a separate set of accessory sites is found: the forth, fifth and seventh most positive kmers match HNF3, whereas the second and third match TAL1. The sixth ranked kmer matched NF1. The eight and tenth ranked kmers match CREB, not reported in John *et al*. (26). In summary, our analysis uncovered most of the accessory factors described in John *et al*. (26), but also identifies novel positive and novel negative binding sites. Further, we demonstrate that these features are predictive, in the sense that these features can be used to accurately classify the positive and negative regions, and are not simply over (or under) represented in one of the sets.

**Figure 4.**
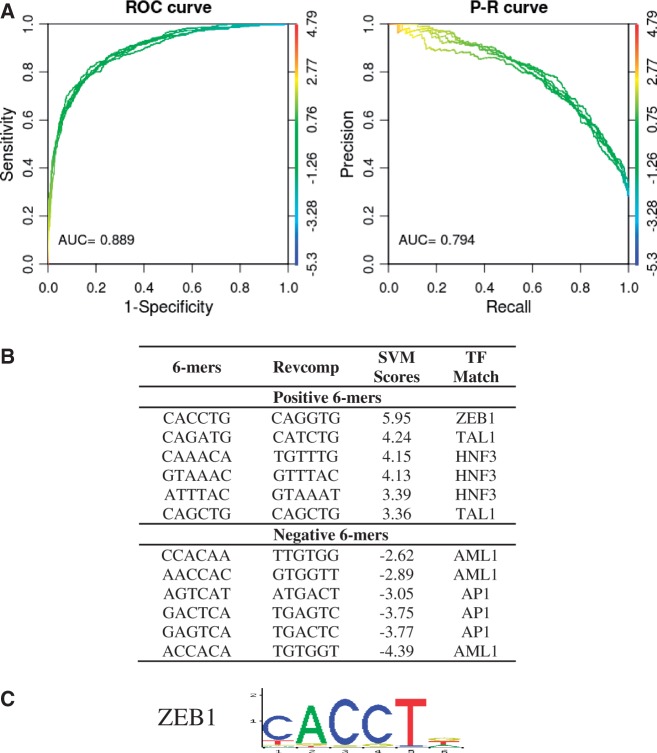
kmer-SVM analysis of sequence determinants of cell-type-specific GR binding. (**A**) ROC and PR curves for a kmer-SVM trained on GR-bound regions in AtT20 cells (positive set) versus GR-bound regions in 3134 Cells (negative set). Default parameters were used; Kernel type = Spectrum, *K* = 6, *C* = 1, *E* = 1e-5, *PSW* = auto. (**B**) The accessory factor binding sites, including ZEB1, TAL1, HNF3, AML1 and AP1, are sufficient to distinguish the distinct sets of GR-bound regions in these two cell lines. The GRBE element is now present in both sets, is not predictive in this context and therefore does not receive a large weight. (**C**) ZEB1 motif from JASPAR database (18) is shown.

We next demonstrate that our kmer-SVM is able to directly distinguish the GR bound regions in 3134 cells from the GR-bound regions in AtT20 cells from DNA sequence. In this case, we do not use random genomic sequence as the negative set, but instead train a kmer-SVM using the AtT20 regions as the positive sequence set, and the 3134 regions as the negative sequence set. The ROC and PR curves are shown in Figure 4a, yielding AUROC of 0.889 and AUPRC of 0.794. Thus, DNA sequence is sufficient to distinguish the cell specific binding of GR. Now, as both sets are bound by GR, the kmer weights shown in Figure 4b do not include the GRBE, as it is present in both sets. The distinguishing features are now binding sites for the GR accessory factors. The kmer CAGGTG (ZEB1), which was negative for 3134 versus random is now the most positive kmer for AtT20 versus 3134. The other positive kmers match the AtT20-specific accessory factors TAL1 and HNF3. The negative weight kmers are the 3134 specific accessory factors AML1 and AP1. This demonstrates that these accessory sequence elements are predictive of the tissue-specific binding of GR because the sequence information in the accessory factor-binding sites is sufficient to distinguish GR binding in these two contexts. We emphasize that this is a stronger statement than simply observing the enrichment of distinct sequence features in the two cases: we further propose the hypothesis that these sequence features are sufficient to specify which GR-binding sites will be occupied in each tissue. This differential occupancy is determined by the presence of binding sites for accessory factors, which can be identified from the kmer weights.

### Prediction of distinct EWS-FLI bound regions in EWS502 and HUVEC cells

Although the previous example showed that binding of a sequence specific TF to different loci in different tissues was predictable from DNA sequence, we now turn to an example where a wild-type and mutant TF were shown to bind distinct regions, and that this differential binding is also predictable from DNA sequence. Most Ewing-Sarcoma tumors harbor a mutation, which creates an oncogenic chimerical EWS-FLI TF by fusing the transactivation domain of EWS to the DNA-binding domain of FLI. Patel *et al*. (27) showed that this chimeric EWS-FLI TF targets different genomic regions in tumor cells and in non-tumor cells, and that additionally the wild-type protein FLI1 binds to largely the same regions as the fusion protein in non-tumor cells. Specifically, the authors assayed binding in the EWS502 cell line (derived from a Ewing Sarcoma tumor) and primary human endothelial cells (HUVEC). They reported a preferential binding for regions containing repeats of the tetranucleotide GGAA by EWS-FLI in both EWS502 and HUVEC cells (although the tumor cell line showed a greater enrichment). Additionally, binding of EWS-FLI in HUVEC cells was shown to be enriched in ETS, AP1 and GATA motifs, but that these accessory motifs were largely absent from the EWS-FLI bound regions in EWS502 cells.

**Figure 5.**
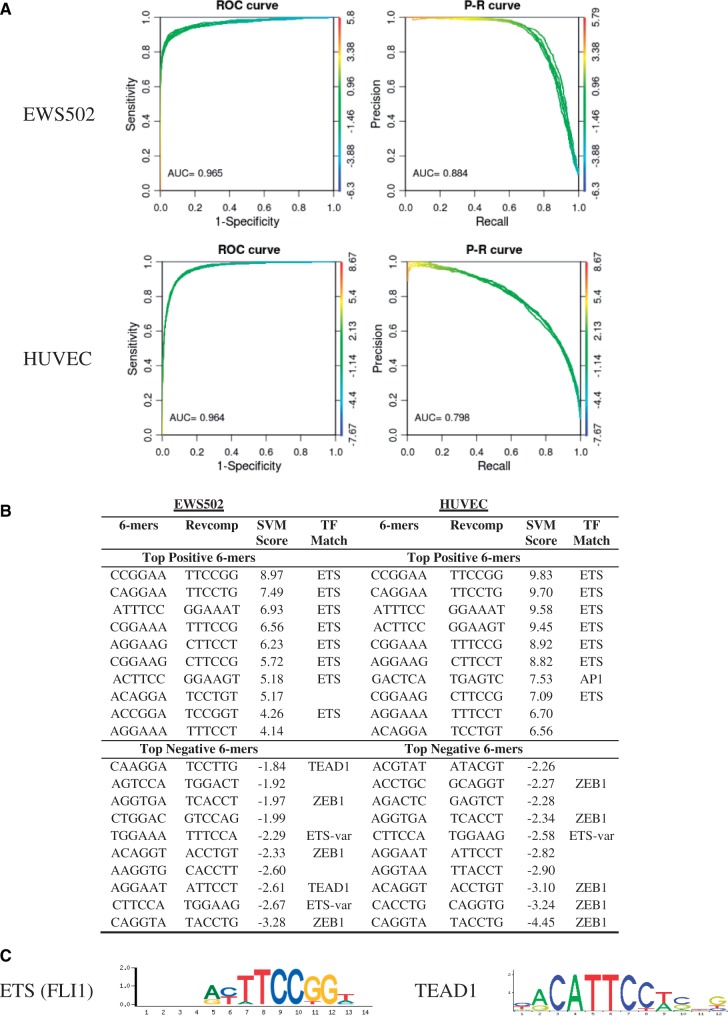
kmer-SVM analysis of EWS-FLI-binding sites.(**A**) ROC and PR curves for a kmer-SVM trained on EWS-FLI-bound regions in EWS502 cells and HUVEC cells versus random genomic sequence. Default parameters were used; Kernel type = Spectrum, *K* = 6, *C* = 1, *E* = 1e-5, *PSW* = auto. (**B**) The 10 most positive, negative 6 mers from EWS502 cells and HUVEC Cells include binding sites the previously reported ETS and AP1 accessory factors, and novel accessory factors TEAD1 and ZEB1. (**C**) ETS (FLI1) from UniPROBE (16) and TEAD1 motif from JASPAR database (18) are shown.

To analyze these data sets, we used as positive sets the ChIP-seq regions in Patel *et al*. (27) bound by EWS-FLI in EWS502 cells and HUVEC cells, and we generated separate 10× negative sets for each cell line. After training the kmer-SVM, in EWS502 cells, the AUROC was 0.965 and AUPRC was 0.884, and in HUVEC cells, the AUROC for this data set was 0.964 and AUPRC was 0.798 (Figure 5a), again showing that the cell line specific binding of the EWS-FLI TF is predictable from primary DNA sequence features. In this case, the training data were optimized for length by the peak-calling algorithm ZINBA (28), which may account for the extremely high classification performance. Another possible factor is that the repeat fraction in these positive sets is relatively high.

Our method finds some motifs common to both cell lines. Positive sequence features reflect both the ETS motif recognized by FLI1 and the repetitive structure reported by Patel *et al*. (27), with the ETS motif GGAA as part of the highest ranked kmers in both cell lines, as shown in Figure 5b. Negative weight kmers are again found to be significant. Kmers that disrupt the repetitive GGAA structure (e.g. TGGAAG) score negatively in both cell lines, but more negatively in EWS502 cells. Notably, many of the most negative kmers for both cell lines contain AGGT, again emphasizing the importance of the absence of ZEB1 or Snail repressor family-binding sites for EWS-FLI binding or function.

Cell line-specific kmers recover the AP1 motif reported in Patel *et al*. (27), and a potentially novel role for TEAD1. The HUVEC specific accessory factor AP1 is found as a high scoring motif in HUVEC cells, but not EWS502 cells. Two highly negative kmers in EWS502 cells correspond to the binding site for TEAD1. TEAD1 has been implicated in tumor suppression and growth control and because the absence of TEAD1 binding sites is predictive of EWS-FLI binding in EWS502 cells, but not HUVEC cells, it is tempting to speculate that TEAD1-binding would disrupt EWS-FLI binding in EWS502 cells, but not in HUVEC cells.

### Kmer-SVM versus PWM

**Figure 6.**
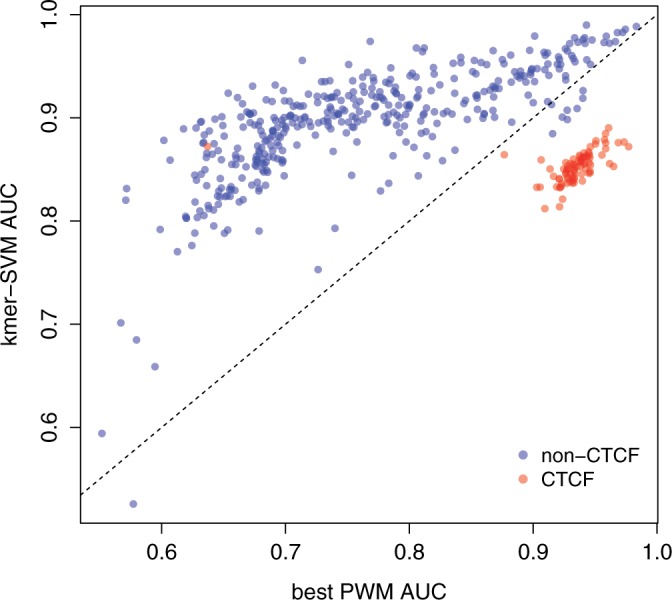
kmer-SVM versus PWM scores. The kmer-SVM AUROCs (*Y*-axis) of the 468 ChIP-seq data sets are compared with the best PWM AUROCs (*X*-axis). Default parameters were used; Kernel type = Spectrum, *K* = 6, *C* = 1, *E* = 1e-5, *PSW* = auto. In general, kmer-SVM is much more accurate than any single PWM with one exception; the CTCF PWM (red circle).

To systematically evaluate our kmer-SVM method on a more exhaustive collection of data, we analyzed all ChIP-seq data sets generated as part of the ENCODE project (29,30). We directly used the 468 sets of peaks generated by ENCODE Uniform processing pipeline (29), after removing any data sets containing <500 peaks (27 sets were excluded by this criterion). We then trained a kmer-SVM model on each set versus an equal size (1×) set of corresponding random genomic regions and calculated the AUROC. As a comparison, we independently calculated the AUROC of each single PWM in a combined database of 890 PWMs, using as predictors the PWM score of the top hit in each region. Figure 6 shows that our kmer-SVM prediction outperforms the best single PWM in almost all cases. The only notable exception is the CTCF PWM (red circles), which is predictive for ChIP on CTCF and members of the cohesin complex (RAD21, SMC3), which are known to co-localize with CTCF (31). CTCF is one of the longest and information rich PWMs and seems to operate in a non-combinatorial manner; therefore, it seems to be relatively unique in that its genomic binding can be predicted with a single PWM. In addition, its long binding site is not handled optimally by the current kmer-SVM model.

## DISCUSSION

We have shown that our kmer-SVM model as offered in this web server is able to find predictive sets of DNA sequence features in several different genomic data sets and can be used to assess and explore the genomic data and generate testable hypotheses for subsequent biological analysis. Using the existing sequence tools and pipeline flow of the Galaxy platform has greatly facilitated the ease of distribution of this tool. The examples we have highlighted earlier in the text, in addition to our previous results on mouse EP300 bound enhancers (3) and melanocyte enhancers (6), emphasize several key benefits of our kmer-SVM analysis. Using our web server, users can find the essential sequence features, which distinguish a set of experimentally determined genomic regions from random sequence, and identify key accessory factors and repressive elements for biological interpretation and follow-up investigations. In addition, users can use the kmer-SVM to score alternative sequence sets or entire genomes to make predictions of the activity of these regions in the relevant context. We hope that release in this form will facilitate open availability and ease of access to the broader research community.

Our web server provides complementarity to existing PWM discovery and scoring tools, including XXmotif (32), MEME (21), SCOPE (33), RSAT (34), RegAnalyst (35) and Amadeus (36). XXmotif operates by attempting to optimize the statistical significance of a given PWM. Specifically, XXmotif develops and then iteratively merges PWMs for motifs until *P*-values cannot be improved. The core of MEME is the use of mixture models, arrived at by means of expectation maximization, to identify motifs. SCOPE uses three different algorithms, separately directed toward identify short non-degenerate motifs, short degenerate motifs and long degenerate motifs, and uses a scoring method to integrate the output from each of these algorithms. SCOPE is a parameter-free program and requires no parameters to be provided by the user. RSAT is a more general toolbox for the analysis of sequence data and uses a tool for motif discovery, which compares the observed occurrence of motifs against the expected presence of that motif, given the distribution of nucleotide occurrence in an organism (37). RegAnalyst uses a series of thresholds applied to the counts of motifs observed in a set of sequences. Amadeus also compares the frequency of the presence of motifs against a background model. In contrast, our web server focuses on finding combinations of sequence features, which are usually more predictive than single motifs, as show in Figure 6.

To the best of our knowledge, there is only one web server available that provides tools (13) with some similarity to our kmer-SVM module. The web server (http://galaxy.raetschlab.org/) offers simple SVM functions including several string kernels as well as other common kernels, such as linear and Gaussian. It also provides means to evaluate prediction performance using ROC and PR curves. This server, however, is mainly intended for general use of SVMs by users with a certain level of computational experience. In contrast, our kmer-SVM is specifically designed to allow biologists with no prior machine learning expertise to quickly and rigorously analyze regulatory sequence data sets. To do so, our tool incorporates modules with functionality required for regulatory sequence analyses and takes into account the specific properties of regulatory elements. First, we modified the spectrum kernel function to account for the fact that TFs bind to double-stranded DNA. We not only count an exact kmer but also count its reverse complement kmer. Redundant kmers are then eliminated from the final feature set to remove the possible bias caused by double counting. Second, we offer a module that generates negative sequence sets to match the distribution of sequence length, GC content and repeat fraction of the corresponding positive sets. This ensures that the SVM classification reflects the most biologically relevant mechanisms. Third, we provide a means to interpret and explain the results by calculating the SVM weights of kmers from a list of support vectors, the primary output of SVM training. Although a useful tool for its intended audience, none of these functionalities aforementioned provided by our web server is available at the Galaxy server at Rätsch’s laboratory.

## SUPPLEMENTARY DATA

Supplementary Data are available at NAR Online: Supplementary Tables 1–6.

## FUNDING

A.S.M. and M.B. were funded in part by the National Institute of Neurological Disease and Stroke [NS062972]; A.S.M. received additional funding from the National Heart Lung and Blood Institute [HL111267]. M.B. was supported by the Searle Scholars Program. Funding for open access charge: NIH NINDS [NS062972].

*Conflict of interest statement.* None declared.
